# Nature-Derived and Synthetic Additives to poly(ɛ-Caprolactone) Nanofibrous Systems for Biomedicine; an Updated Overview

**DOI:** 10.3389/fchem.2021.809676

**Published:** 2022-01-19

**Authors:** Shahin Homaeigohar, Aldo R. Boccaccini

**Affiliations:** ^1^ School of Science and Engineering, University of Dundee, Dundee, United Kingdom; ^2^ Institute of Biomaterials, Department of Materials Science and Engineering, University of Erlangen-Nuremberg, Erlangen, Germany

**Keywords:** polycaprolactone, additive, nanocomposite, blend, core-shell, wound dressing, tissue engineering

## Abstract

As a low cost, biocompatible, and bioresorbable synthetic polymer, poly (ɛ-caprolactone) (PCL) is widely used for different biomedical applications including drug delivery, wound dressing, and tissue engineering. An extensive range of *in vitro* and *in vivo* tests has proven the favourable applicability of PCL in biomedicine, bringing about the FDA approval for a plethora of PCL made medical or drug delivery systems. This popular polymer, widely researched since the 1970s, can be readily processed through various techniques such as 3D printing and electrospinning to create biomimetic and customized medical products. However, low mechanical strength, insufficient number of cellular recognition sites, poor bioactivity, and hydrophobicity are main shortcomings of PCL limiting its broader use for biomedical applications. To maintain and benefit from the high potential of PCL, yet addressing its physicochemical and biological challenges, blending with nature-derived (bio)polymers and incorporation of nanofillers have been extensively investigated. Here, we discuss novel additives that have been meant for enhancement of PCL nanofiber properties and thus for further extension of the PCL nanofiber application domain. The most recent researches (since 2017) have been covered and an updated overview about hybrid PCL nanofibers is presented with focus on those including nature-derived additives, e.g., polysaccharides and proteins, and synthetic additives, e.g., inorganic and carbon nanomaterials.

## Introduction

Thanks to its promising biocompatibility and low biodegradation rate, poly (ɛ-caprolactone) (PCL) is among the most widely applied synthetic polymers in biomedicine (Mochane et al., 2019). PCL was developed for the first time in the 1930s by the Carothers group (Natta et al., 1934). Its commercialization was aimed very soon due to the need of biodegradable synthetic polymers, i.e., those that could be degraded *in vivo* by cells and microorganisms (Woodruff and Hutmacher, 2010). Despite early popularity of PCL, it was rapidly outstripped by biodegradable polymers like polylactic acid (PLA) and polyglycolic acid (PGA), due to an emerging need to obtain degradable drug delivery systems able to release cargo in a few days or a few weeks with the carriers totally bioresorbed in 2–4 months after application (Woodruff and Hutmacher, 2010). On the other hand, the medical device market was looking for replacement of metal implants (e.g., nails, screws, plates, among others) by their biodegradable versions. In this regard, PCL could not be applicable due to its insufficient mechanical properties for such high load bearing applications. Most importantly, it was proven that the polymers with a high resorption rate are less problematic in terms of biological responses compared to those with a lengthy degradation process (e.g., PCL with a 3–4 year degradation cycle). As a result, PCL was ignored for around 2 decades (Woodruff and Hutmacher, 2010). Afterwards, during the 1990s and 2000s, coinciding with a revolution in regenerative medicine with tissue engineering, interest in PCL as a biomedical material resurged. Such renewed interest in PCL originated from its appropriate viscoelastic and rheological properties compared to other biodegradable polymers, rendering its manufacturing simple and its integration into a variety of scaffolds feasible (Lee et al., 2003; Huang et al., 2007; Luciani et al., 2008; Marrazzo et al., 2008).

PCL can be synthesized via ring-opening polymerization (ROP) of ɛ-caprolactone involving a diverse range of cationic, anionic, and co-ordination catalysts. Additionally, it can be prepared through the free radical ROP of 2-methylene-1-3-dioxepane (Pitt, 1990). In order to catalyze the ROP, non-toxic, efficient catalysts, e.g., stannous (II) 2-ethylhexanoate and to govern PCL’s molecular weight, low molecular weight alcohols are typically employed (Woodruff and Hutmacher, 2010). Other than ROP, PCL can be also synthesized through polycondensation of 6-hydroxycaproic (6-hydroxyhexanoic) acid. ROP is advantageous over the polycondensation technique in terms of production of a less polydisperse polymer with a higher molecular weight (Labet and Thielemans, 2009; Siddiqui et al., 2018).

PCL is a hydrophobic and semi-crystalline polymer (depending on its molecular weight, crystallinity varies). PCL is dissolved in a variety of solvents and is readily melted at low to moderate temperatures (59–64°C). Moreover, it can be properly blended with many polymers and biopolymers, thereby being proposed for diverse biomedical applications (Chandra and Rustgi, 1998; Okada, 2002; Nair and Laurencin, 2007). Such features of PCL and its derivatives (blends, composites, and copolymers) led to their wide implementation in drug delivery systems, during the 1970s and 1980s (Woodruff and Hutmacher, 2010). Compared to other available biodegradable polymers, PCL could offer distinct advantages, thereby surpassing other candidates for biomedical applications. For instance, customizable degradation rate and mechanical properties, simple shaping and production that could allow for creation of pore sizes suitable for tissue ingrowth, and the possibility of drug delivery in a controlled manner are important merits of PCL based systems (Woodruff and Hutmacher, 2010). Additionally, PCL could be functionalized by inclusion of various functional groups, rendering it adhesive, hydrophilic, and biocompatible with proper cell-matter interactions. Taking into account the slower degradation rate of PCL compared to its counterparts such as PGA and poly d,l-lactide acid (PDLA), it could be employed in drug delivery systems with a life span of over 1 year and in commercial suture materials (Maxon™) (Woodruff and Hutmacher, 2010). The caprolactone polymers including PCL are mainly degraded via hydrolysis. The first step of hydrolysis involves diffusion of water molecules into the polymer. Subsequently, the polymer undergoes arbitrary fragmentation and eventually major hydrolysis that is intensified by metabolism and phagocytosis. In general, hydrolysis is governed by the size (molecular weight), crystallinity, and hydrophilicity of the polymer, and hydrolysis rate is modulated by temperature and pH of the nearby environment (Lacoulonche et al., 1999; Kim et al., 2012). The hydrolysis (degradation) of PCL solely produces caproic acid, that is a non-toxic metabolite, either removed from the body through urinary secretion or metabolized via the citric acid cycle (Kweon et al., 2003). The bioresorption time of PCL spans from several months to more than 1 year, and can be controlled through copolymerization and hybridization with additives and blending materials (Cohn and Hotovely Salomon, 2005). PCL has also been shown to undergo enzymatic degradation by lipase-type enzymes such as *pseudomonas* lipase (PS) within a short time of 4 days (Gan et al., 1997). Such degradation process is notably faster than hydrolytic degradation that could take up to several years (Li and Vert, 1999). Among the lipase-based enzymes, Aspergillus sp. lipases, which are in fact a type of fungal lipases, have been appealing in recent years due to their industrial application (Contesini et al., 2010; Wang H. et al., 2017). As a result, the degradation behaviour of PCL mediated by Aspergillus sp. Lipases under various environmental conditions (pH, temperature, etc.) has been precisely studied (Hermanová et al., 2012; Hermanová et al., 2013). In this regard, PCL hydrophobicity has been proven to be a hindering factor against rapid enzymatic degradation (Wang H. et al., 2017). Thus, surface treatment of PCL via aminolysis, hydrolysis, laser ablation, etc., and blending or copolymerization of PCL with hydrophilic polymers, e.g., PEG can be considered as solutions to expedite the enzymatic degradation (Wang H. et al., 2017).

**FIGURE 1:**
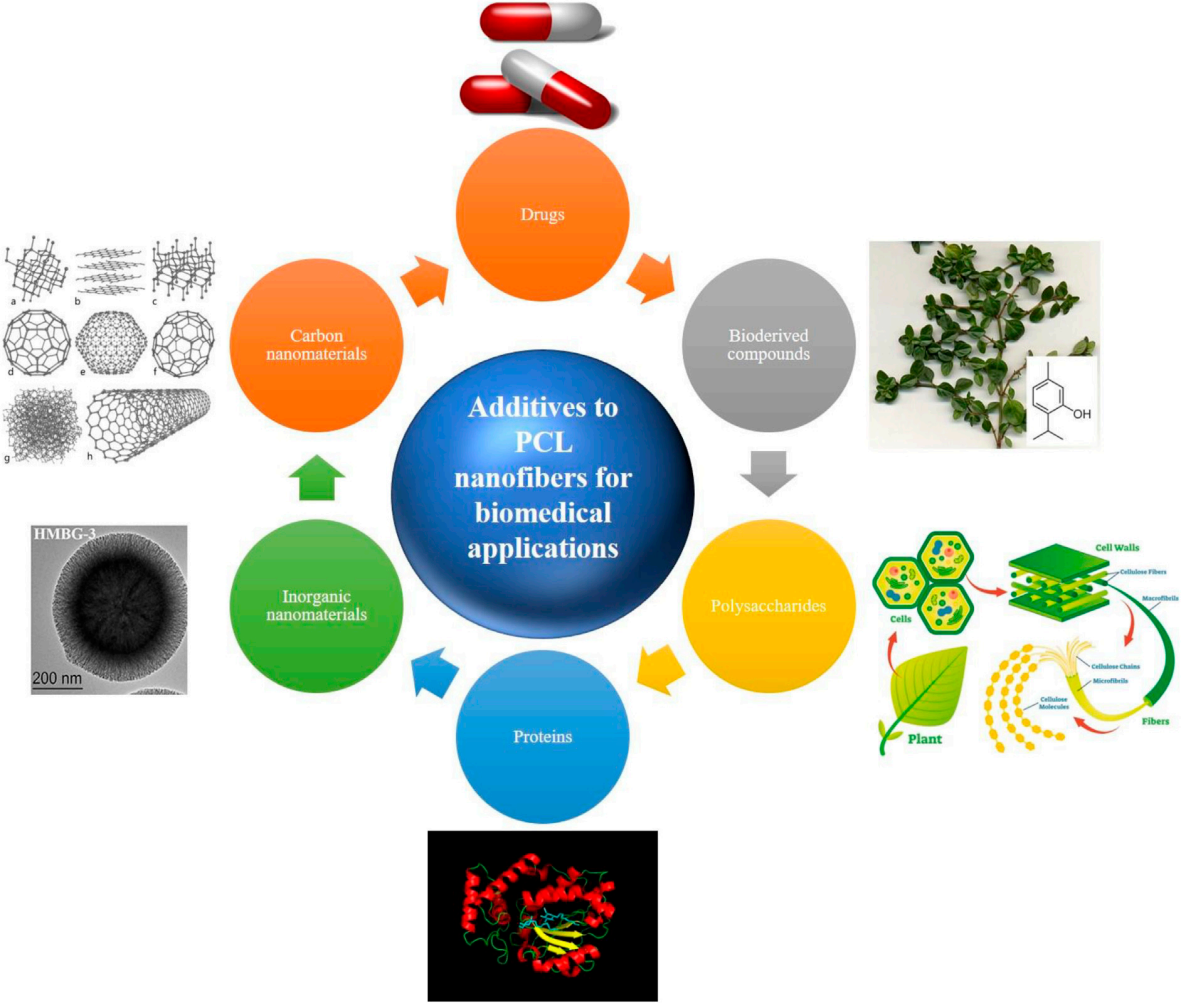
Various classes of additives employed to reinforce biological and physicochemical properties of PCL nanofibers. The used images have been reproduced with permission (inorganic nanomaterials (Wang Y. et al., 2019); CC BY licence, proteins (upload.wikimedia.org/wikipedia/commons/9/95/Sec14p_Protein_Figure.png; CC BY-SA 4.0), polysaccharides (researchoutreach.org/articles/cellulose-nanodefects-key-biofuels-biomaterials-future; CC BY-NC-ND 4.0), bioderived compounds (commons.wikimedia.org/wiki/File:Thyme-thymol; CC BY-SA 4.0), drugs (freepngimg.com/png/26873-pills-photos; CC BY-NC 4.0), and carbon nanomaterials (upload.wikimedia.org/wikipedia/commons/f/f8/Eight_Allotropes_of_Carbon.png; CC BY-SA 3.0).

PCL can be processed in different physicochemical ways to produce biomedical systems at low cost compared to other aliphatic polyesters. Among all the production techniques, electrospinning is the most widely studied one for development of PCL nanofibrous materials for wound dressing and tissue engineering. The applicability of PCL nanofibers can be extended and their properties can be optimized by employment of benign solvents that assure eco-friendly processing (Liverani et al., 2018; Homaeigohar et al., 2021a) and/or inclusion of additives that raise hydrophilicity, bioactivity, and mechanical stability. In this review, as schematically shown in Figure 1, we introduce newly (since 2017) proposed additives and blending materials for PCL electrospun nanofibers that have found application in biomedicine. It is worthy to note that in many studies a combination of various types of additives, e.g. nature derived compounds alongside a drug, has been employed. This feature makes distinct categorization of additives challenging and therefore the overlap of different classes is inevitable.

## Reinforcing and Hydrophilizing Additives

Given the direct impact of nanofiber physicochemical properties on the cell-material interactions, it is crucial to optimize surface hydrophilicity and mechanical properties of the PCL nanofibrous biomaterials. The resilience and mechanical strength of nanofiber materials have been proven to affect *in vitro* cell behaviour including migration, proliferation, and differentiation, and also cell shape (Carnegie and Cabaca, 1993; Zhang et al., 2005). Generally, cells are optimally extended and spread on a resilient, flexible underlying material. They exert a tensile force on the substrate, particularly while they migrate. Cells sense the elasticity of the surface they are migrating on by using their integrins, that perform as mechanotransducers. In contrast to a pliable surface, cells have to apply an extremely large tension force to migrate on a notably stiff or rigid material, thus adopting a less extended shape (Gunn et al., 2005; Homaeigohar et al., 2019). Therefore, developing a mechanically compatible PCL nanofiber material, depending on the target tissue and biomedical purpose profoundly matters. To achieve this goal, a variety of additives have been proposed that raise mechanical properties of PCL nanofibers, particularly in terms of tensile strain, flexural strain, elastic modulus, tensile stress, and thermomechanical strength (Gönen et al., 2016; Adeli-Sardou et al., 2019; Nazeer et al., 2019; Zanetti et al., 2019). In this regard, various fillers such as nanosilicates (Wang et al., 2018), graphene, cellulose nanocrystals, Ag nanoparticles (Leonés et al., 2020), among others, have been incorporated into PCL nanofibers to confer them with improved mechanical properties. Additionally, PCL has been blended with natural (Ren et al., 2017) or synthetic polymers (Nadim et al., 2017) to create mechanically robust blend PCL nanofibers for biomedical applications. Table 1 tabulates several examples of PCL nanofiber materials that have been mechanically reinforced by inclusion of fillers and blending agents.

**TABLE 1:** Mechanically stabilized PCL-based nanofibers for biomedical applications (studies reported after 2017).

Reinforcing additive	Nanofiber carrier material	Additive loading method	Optimized mechanical property	Target application	References
Nanosilicate	PCL	Blend electrospinning	Tensile strength (50.17 Vs. 4.53 MPa for PCL nanofibers)	Bone tissue engineering	Wang et al. (2018)
Gel	PCL	Blend electrospinning	Elastic modulus (105 Vs. 40 MPa for PCL nanofibers)	Bone tissue regeneration	Ren et al. (2017)
CNC	PCL	Blend electrospinning	Tensile strength (3 Vs. 1 MPa for PCL nanofibers)	---	Leonés et al. (2020)
PCL grafted GO	PCL	Blend electrospinning	Tensile strength (14.32 Vs. 5.4 MPa for PCL nanofibers)	---	Bagheri and Mahmoodzadeh (2020)
CNT	PCL	Blend electrospinning	Tensile strength (8.84 Vs. 3.13 MPa for PCL nanofibers)	---	Wang et al. (2017b)
Cartilage-derived ECM	PCL	Blend electrospinning	Tensile strength (3.38 Vs 2.15 MPa for PCL nanofibers)	Cartilage regeneration	Feng et al. (2020)
TPU	PCL	Blend electrospinning	Circumferential tensile strength (7.5 Vs. 6 MPa for PCL nanofibers)	Vascular grafts	Mi et al. (2018)
Nanofibrillated CS	PCL	Blend electrospinning	Tensile strength (6 Vs. 2.57 MPa for PCL nanofibers)	---	Fadaie et al. (2018)

CNC, cellulose nanocrystal; CNT, carbon nanotube; CS, chitosan; ECM, extracellular matrix; Gel, gelatine; GO, graphene oxide; TPU, thermoplastic polyurethane.

Surface hydrophilicity of nanofiber materials has been shown to be a decisive factor in cell-material interaction, particularly on cell adhesion. One important reason is the increased protein adsorption on the surfaces with moderate hydrophilicity (Lee et al., 1998). The improved adsorption of cell-adhesive serum proteins such as vitronectin and fibronectin can highly affect cell adhesion, growth, and morphology (van Wachem et al., 1987; Underwood and Bennett, 1989; Steele et al., 1995). PCL nanofibers are inherently hydrophobic and do not expose polar functional groups on the surface. To address this shortcoming and to improve wettability thus cell interactivity of PCL nanofibers, they have been either surface coated/blended with hydrophilic natural polymers, e.g., collagen (Col), gelatine (Gel), chitosan (CS), etc., or incorporated with polar inorganic nanofillers made of, e.g., Ag and TiO_2_ and bio-derived compounds, e.g., *lawsone*, *Nigella Sativa,* etc*.* (Wang H. et al., 2017; Ghosal et al., 2017; Ren et al., 2017; Adeli-Sardou et al., 2019; Sharifi et al., 2020). In this regard, recently, Li et al. (2021) developed a Col/PCL nanofiber wound dressing with superior wettability and mechanical stability. Col, as a natural (biological) polymer and the main constituent of ECM, has been extensively employed for the repair of damaged skin tissues and wound healing, thanks to its outstanding biocompatibility and inferior antigenicity (Rho et al., 2006; Kubow et al., 2015). However, Col is mechanically unable to support large colonies of cells and is rapidly degraded by enzymes (Engelhardt et al., 2011). Such shortcomings discourage the researchers to benefit from Col in practical wound healing applications (Li et al., 2021). These limitations can be addressed by hybridizing Col with mechanically robust synthetic polymers such as PCL. On the other hand, inherent hydrophobicity issue of PCL, adversely impacting its biological properties including cell adhesion and proliferation, can be resolved (Li et al., 2021). As reported by Li et al. (Li et al., 2021), Col/PCL nanofibers show improved hydrophilicity, reflected in a reduced water contact angle to 40° from 88°, thus offering a high exudate uptake capacity over the course of the wound healing process. Additionally, the most optimum hydrophilicity seen for Col/PCL (1:1) nanofibers brings about raised cell adhesion.

Table 2 tabulates a variety of PCL nanofiber systems that have been hydrophilized by addition of fillers and blending agents.

**TABLE 2:** Hydrophilized PCL-based nanofibers for biomedical applications (studies reported after 2017).

Hydrophilic additive	Nano/microfiber carrier material	Additive loading method	Reduced water contact angle (from–to)	Target application	References
Lawsone	PCL/Gel core-shell	Blend electrospinning	116°–111°	Skin tissue regeneration	Adeli-Sardou et al. (2019)
Gel	PCL	Blend electrospinning	127°–57°	Bone tissue regeneration	Ren et al. (2017)
NS	PCL	Blend electrospinning	107°–46°	Wound healing	Sharifi et al. (2020)
CS	CNT/PCL	Surface coating via immersion of the nanofibers in a CS solution	128.4°–14.7°	---	Wang et al. (2017b)
PEO	PCL	Blend electrospinning	115.35°–10.86°	Drug delivery	Eskitoros-Togay et al. (2019)
Col	PCL	Coating the nanofibers	88°–40°	Skin tissue engineering	Ghosal et al. (2017)
TiO_2_	PCL	Blend electrospinning	88°–80°	Skin tissue engineering	Ghosal et al. (2017)
Nano hydroxyapatite	PCL	Blend electrospinning	104°–98°	Bone tissue regeneration	Perumal et al. (2020)

Col, collagen; NS, *nigella sativa*.

## Therapeutic Additives

### Drugs

Thanks to an enhanced therapeutic effect and a lower toxicity, localized drug delivery with a steady, controlled rate is preferred over systemic drug administration (Zeng et al., 2003). Therefore, development of efficient drug delivery systems has been extensively researched during the past decades. In this regard, the drug carrier material needs to be properly biodegradable, not only shielding the drug against aggressive biological environments, but also allowing the drug to be delivered in a tailored manner (Karuppuswamy et al., 2015).

Electrospun nanofibers have shown promising applicability for topical drug delivery, for example assuring a steadier drug release compared to cast films (Kenawy et al., 2002). This feature originates from the high surface area of nanofibers and interconnectivity of the nanofibrous mat, allowing high permeability of drug molecules and other therapeutic agents such as peptides, proteins, and antibodies, already incorporated into or loaded onto the nanofibers (Son et al., 2014). In this regard, various biodegradable polymers including PCL have been investigated to create nanofibrous drug delivery systems for wound healing and tissue engineering. It has been shown that tissue engineering scaffolds loaded with therapeutic agents, e.g. antibacterial, anti-inflammatory, and anti-cancer synthetic or natural drugs, offer a higher tissue regeneration efficiency (Jayakumar et al., 2011).

**FIGURE 2:**
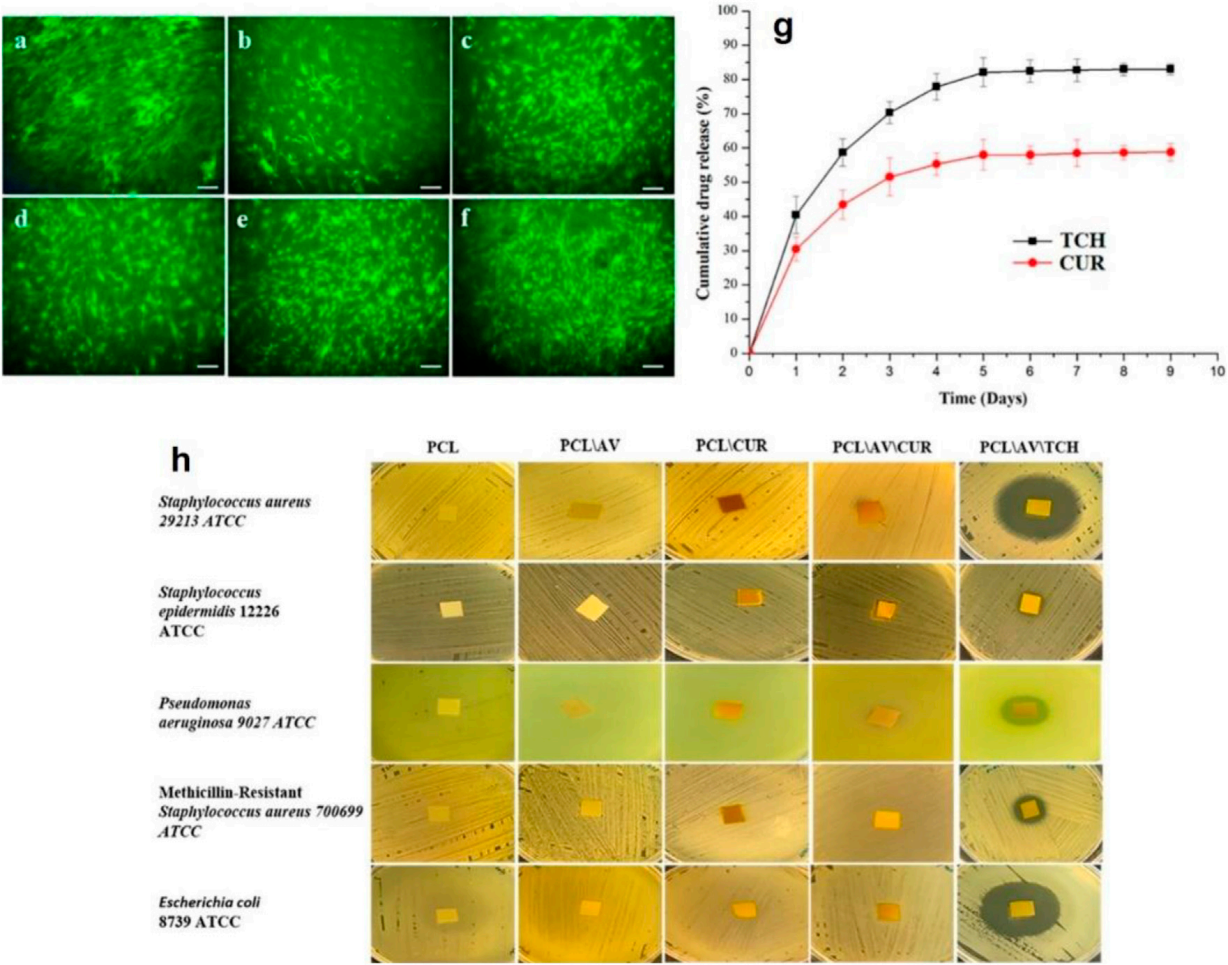
Live fibroblast cells present on **(A)** control, **(B)** PCL nanofibers, **(C)** AV/PCL nanofibers, **(D)** CUR/PCL nanofibers, **(E)** AV/CUR/PCL nanofibers, and **(F)** AV/TCH/PCL nanofibers (scale bars represent 50 µm). **(G)** TCH and CUR release profiles of the AV/CUR/PCL and AV/TCH/PCL nanofibers over a 9-days time period. **(H)** Antibacterial activity of the drug loaded PCL nanofiber systems. The images were reproduced under CC BY licence (Ezhilarasu et al., 2019), Copyright 2019, MDPI.

Tetracycline (TC) is an antibiotic that is placed within the World Health Organization (WHO)׳s list of Essential Medicines, which includes the vital medications necessary for any basic health system (Karuppuswamy et al., 2015). As a proven fact, steady release of TC facilitates the proliferation of fibroblasts and osteoblasts and properly limits infections, when used as bone and skin ointments (Karuppuswamy et al., 2015). PCL nanofibers have been employed as a TC hydrochloride (TCH) carrier for skin tissue engineering. For example, synergistically, alongside aloe vera (AV) and curcumin (CUR), the TCH-AV-CUR/PCL nanofibers are able to induce a proper fibroblast cell viability, as shown in Figures 2A–F (Ezhilarasu et al., 2019). Compared to the composite PCL nanofibers, the neat ones induced an insignificant proliferation rate most likely due to lack of cell recognition moieties that could encourage cell-nanofiber interaction and cell adhesion. Figure 2G shows how steady CUR and TCH are released form the PCL nanofibers over a 9-days time period. However, surface residence of some part of the loaded drugs brings about an initial burst release of 30.1% (CUR) and 41.5% (TCH). Moreover, the appropriate release profile of TCH indicated a favourable antibacterial performance, as shown in Figure 2H, represented by formation of clear zones around the TCH-AV/PCL nanofiber mat.

Oxytetracycline hydrochloride (OTC) is a member of the TC antibiotics family that plays a bacteriostatic role via hampering the synthesis of bacterial protein. These antibiotics are lethal to an extensive range of both Gram-negative and Gram-positive bacteria (Chopra and Roberts, 2001). TC antibiotics are excreted in the gingival crevicular fluid (GCF) and firmly adsorb to the tooth surface. As a result, TC antibiotics are preserved in the oral cavity for a long time (Baker et al., 1983), and can be largely applied for periodontal disease treatment. PCL nanofibers have been considered as a carrier of OTC for the mentioned application (Dias et al., 2019). Alongside OTC, ZnO nanoparticles have also been employed to further support the system’s antibacterial activity. ZnO’s antibacterial effect is fulfilled through three main mechanisms: 1) generation of reactive oxygen species (ROS) on the ZnO surface (Lakshmi Prasanna and Vijayaraghavan, 2015; Sirelkhatim et al., 2015), 2) disintegration of cell (bacteria) membrane upon contact with the ZnO nanoparticles and subsequent interaction between the nanoparticles and the cell content (Joe et al., 2017), and 3) release of bactericidal Zn^2+^ ions from ZnO when subjected to the aqueous medium (Joe et al., 2017). In addition to an enhanced antibacterial activity, OTC/PCL nanofibers containing ZnO nanoparticles were shown to offer a steadier OTC release behaviour, most likely due to electrostatic interaction between the additives, i.e. the drug and the nanoparticles. Despite entrapment of OTC within PCL nanofibers, hydrolysis of the nanofibers eventually leads to the total release of the antibiotic into the periodontal pockets after 5 days. While an enhanced antibacterial effect for the system comprising both OTC and ZnO was expected, the challenging release of OTC led to a weaker antibacterial efficiency (97.5%) compared to the OTC/PCL nanofibrous system (98%). On the other hand, while ZnO is beneficial in terms of antibacterial activity, it might also induce cytotoxicity. According to a study by Cho et al. (Cho et al., 2013), among ZnO, CuO, SiO_2_, and Co_3_O_4_ nanoparticles, ZnO and CuO showed the highest level of cytotoxicity *in vitro* and could impose acute lung inflammogenicity *in vivo*.

Table 3 tabulates the studies dealing with drug incorporated PCL and PCL blend nanofibers carried out after 2017.

**TABLE 3:** Drug loaded PCL-based nanofibers for biomedical applications (studies reported after 2017).

Drug	Nano/microfiber carrier material	Drug loading method	Therapeutic function	Target application	References
TCH	PLA/Gel/PCL	Blend electrospinning	Antibacterial effect	Dental implant coating	Shahi et al. (2017)
TCH	GO/Gel/PCL	Blend electrospinning	Antibacterial effect	Neural tissue engineering	Heidari et al. (2019)
TNZ	Chitosan/PCL	Blend electrospinning	Antibacterial effect	Treatment of periodontitis	Khan et al. (2017)
BSA	PLA/PCL	Blend electrospinning	Drug model	---	Herrero-Herrero et al. (2018)
Ketoprofen	PCL/Gel	Blend electrospinning	Anti-inflammatory effect	Wound dressing	Basar et al. (2017)
Cefazolin	PCL	Blend, emulsion, and co-axial electrospinning	Antibacterial effect	---	Radisavljevic et al. (2018)
Phenytoin	PVA (core)/PCL (shell)	Core-shell electrospinning	Proliferative effect	Tissue regeneration and wound healing	Mohamady Hussein et al. (2021)
Doxorubicin	PAMAM-PCL/PCL	Blend electrospinning	Chemotherapeutic effect	A delivery system of anticancer drugs	Bala Balakrishnan et al. (2018)
Curcumin	PEDOT NP/PCL	Blend electrospinning	antibacterial, antiviral, antifungal, and anticancer	---	Puiggalí-Jou et al. (2018)
Ibuprofen	PCL	Blend electrospinning	Anti-inflammatory effect	---	Kamath et al. (2020)
Doxycycline	PEO/PCL	Blend electrospinning	Antibacterial effect	---	Eskitoros-Togay et al. (2019)
TC/β-cyclodextrin	PCL	Blend electrospinning	Antibacterial effect	Treatment of periodontitis	Monteiro et al. (2017)

BSA, bovine serum albumin; NP, nanoparticle; PLA, polylactide acid; PEDOT, poly (3,4-ethylenedioxythiophene); PEO, polyethylene oxide; PAMAM, poly (amido-amine); PVA, polyvinyl alcohol; TNZ, tinidazole.

### Bioderived Therapeutic Compounds

In addition to drugs, natural substances or bio-derived agents are also considered as a potential therapeutic additive to the PCL nanofibers for biomedical applications. For instance, resveratrol (RSV) is a natural substance with therapeutic effects against periodontal disease. It shows anti-oxidative and anti-inflammatory effects that alleviate the adverse consequences of the periodontal disease such as lower generation of NO (Rizzo et al., 2012), excessive expression of vascular endothelial growth factor (VEGF) by human gingival fibroblasts, and reduced permeability of vessels (Núñez et al., 2010). Furthermore, RSV hampers the large production of chemokines, inflammatory cytokines, and the factors driving leucocyte differentiation (Fordham et al., 2014). Performing as a blocker for the aryl-hydrocarbon receptor, RSV positively influences periodontal tissue regeneration (Singh et al., 2000) and notably declines bone tissue loss (Casati et al., 2013). Despite the mentioned merits, RSV is unstable under *in vivo* conditions thanks to its undesired biopharmaceutical characteristics such as poor solubility, fast metabolism, and insufficient chemical resistance. Accordingly, there is a need to creation of a carrier system that tackles the biopharmaceutical challenges and thereby maximizes the prophylactic and therapeutic capacity of RSV (Amri et al., 2012). In this regard, nanofibers are employed to develop state of the art drug delivery systems that can hold RSV and similar bioderived agents and release them in a tailored manner through engineering of their composition and morphology (Zamani et al., 2010; Zupančič et al., 2015). Additionally, having an extensive surface-to-volume ratio, nanofibers tend to stick to the periodontal pocket tissue and due to their specific morphology allow for penetration of the gingival crevicular fluid (GCF) through them. This feature declines the possibility of removal of the delivery system from the periodontal pockets, in contrast to less porous chip or film shaped counterparts (Jain et al., 2008; Pelipenko et al., 2015). One of the most suitable materials for the synthesis of RSV carrier nanofibers is PCL. In this regard, Zupancic et al. (Zupančič et al., 2015) included RSV in the PCL nanofibers and validated its applicability for periodontal disease treatment. According to their study, RSV was released steadily from PCL nanofibers. However, at the lower RSV concentrations, release took place at a slower rate owing to hydrophobic interaction and hydrogen bonding between RSV and PCL.

Plant polyphenols include a variety of compounds with several phenolic functionalities and are synthesized by the majority of higher plants as secondary metabolites. Such compounds have been proven to show chemopreventive, cardioprotective, and neuroprotective properties (Havsteen, 2002; Williams et al., 2004). More importantly, plant polyphenols could block the formation pathway of malignant tumors, through inactivating carcinogen and provoking the carcinogen-detoxifying systems (Birt et al., 2001). As an example, Epigallocatechin-3-O-gallate (EGCG), a well-known polyphenolic compound commonly found in green tea, shows a preventive role against cancer (Kim et al., 2012). This compound hinders formation of tumor *in vivo* and offers anticancer effects *in vitro* (Moyers and Kumar, 2004), when altering the release (expression) of the main molecules involved in transcription and cell cycle progression. It also triggers the mitogen-driven protein kinase cascade and eventually inhibits telomerase (Nam et al., 2001). Caffeic acid (CA), a plentiful hydroxycinnamic acid, is another plant polyphenol compound typically found in several plant-originated materials (Caccetta et al., 2000). Dietary CA can be easily absorbed through gut and enters into the blood plasma with a concentration at micromolar levels. CA induces various anticarcinogenic, antimutagenic, anti-inflammatory, and antioxidative effects (Chung et al., 2006). As reported by Chung et al. (Chung et al., 2004), CA can potentially drive apoptosis in the cancer cell lines and as a result it inhibits tumor growth *in vivo*. Benefitting from the anticancer properties of CA and EGCG, Kim et al. (Kim et al., 2012) developed CA and EGCG incorporated PCL nanofibers to assure a long term cancer treatment following surgery. No burst release of polyphenols was recorded after loading into the PCL nanofibers and the plant-based compounds were released in a controlled manner. As a result, H_2_O_2_ was generated that could optimally activate caspase-3 of gastric cancer cells and eventually induce cell apoptosis.

Table 4 tabulates the main bioderived compounds that have been investigated as an additive to PCL nanofibers for biomedicine.

**TABLE 4:** Bioderived compound loaded PCL nanofibers for biomedical applications (studies carried out since 2017).

Bioderived compound	Nano/microfiber carrier material	Loading method	Therapeutic function	Target application	References
*Carvacrol*	PCL	Blend electrospinning	Antimicrobial and anti-oxidative effects	---	Tampau et al. (2017)
*Thymol*	Mesoporous silicon dioxide NP/PCL	Physical blending and electrospinning	Antibacterial activity	Wound dressing	Gámez et al. (2020)
*Spirulina*	Alginate/PCL	Blend electrospinning	Anti-inflammatory and anti-oxidative effects	Wound dressing	Kim et al. (2018)
Calendula officinalis/Gum Arabic/Zein	PCL	Suspension, and two nozzle electrospinning	Calendula officinalis: blood coagulation activity, antibacterial, antiseptic, antiviral, antifungal, anti-inflammatory, free radical inhibitors, antioxidant activity	Skin regeneration	Pedram Rad et al. (2019)
			Gum Arabic: hemostatic, antibacterial and antioxidant activities		
			Zein: antioxidative activity		
*Nigella sativa*	PCL	Blend electrospinning	Antibacterial activity	Wound healing	Sharifi et al. (2020)
*Wattakaka volubilis*	PCL	Blend electrospinning	Bone regeneration effect	Bone and cartilage tissue engineering	Venugopal et al. (2019)
*Tridax procumbens*	PCL	Surface deposition	Antibacterial activity	Wound healing	Suryamathi et al. (2019)
*Peppermint essential oil*	PCL	Blend electrospinning	Antibacterial activity	Wound healing	Unalan et al. (2019)
*Inula graveolens*	PCL	Blend electrospinning	Antimicrobial and anti-oxidative effects	Not reported	Al-Kaabi et al. (2021)

## Bioactive Additives

### Blending Bioagents (Polysaccharides)

**FIGURE 3:**
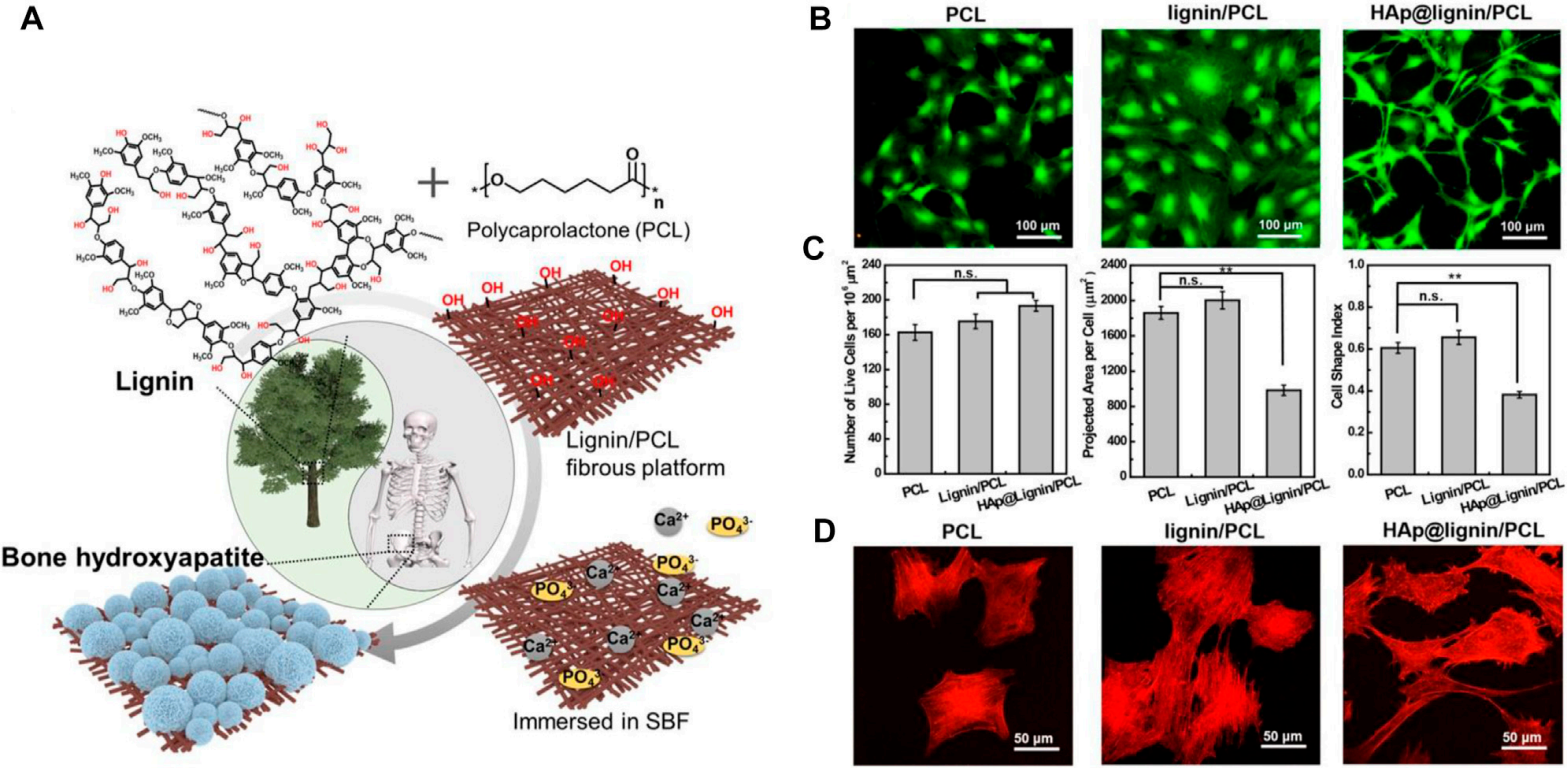
**(A)** Schematic diagram illustrating the biomineralization process taking place on the surface of lignin/PCL nanofibers thanks to abundance of hydroxyl groups that facilitate nucleation and growth of the SBF’s calcium and phosphate ions. *In vitro* biocompatibility test of the HA-lignin/PCL nanofibers, validated through **(B)** LIVE/DEAD assay on the MC3T3-E1 cells after 48 h incubation in the presence of the nanofibers, **(C)** live cell density/10^6^ μm^2^ (left), projected cell area (middle), Cell shape index (CSI) analysed based on the fluorescent images (right). The CSI indicates how circular a cell is. It ranges from 0 (linear) to 1 (circle) (**p < 0.01, n. s not significant). **(D)** Cytoskeleton configuration of the MC3T3-E1 cells present on the nanofibers. Reproduced with permission. (Wang D. et al., 2019), Copyright 2019, American Chemical Society.

Natural polymers, e.g., chitosan, alginate, and lignin, can play a structural/supportive role for PCL nanofibers raising their functionality. Lignin, that is commonly found in the vascular plants’ cell walls, is ranked second among organic molecules in terms of abundancy (Wang D. et al., 2019). It comprises phenylpropanes and monolignols in different ratios depending on the type of the plant source and structurally is a randomly cross-linked polymer (Wang D. et al., 2019). Each year the pulp industry produces over 70 million tons of lignin by-products, thereof only 2% is commercialized as a constituent of adhesives, surfactants and dispersants for rubbers and plastics (Norgren and Edlund, 2014). Lignin is highly durable, thermally resistant, and biocompatible. Moreover, it shows desirable antibacterial activity and protects the cells against oxidative stresses. Therefore, it is potentially applicable for various healthcare purposes (e.g., as a drug carrier material for cancer treatment, antibacterial material, and free-radical scavenger) (Naseem et al., 2016). Nevertheless, lignin is quite brittle, possesses a largely intricate three-dimensional (3D) structure, and is incompatible with apolar polymers (Sen et al., 2015). Such shortcomings have motivated researchers to develop lignin biomaterials with proper elasticity and homogeneity. In this regard, one promising solution is blending of lignin with a polar polymer. The presence of phenolic hydroxyl group in lignin that can properly form hydrogen bonds with the polymer’s electron accepting groups such as carbonyl and ether groups, enable creation of a homogenous lignin/polymer blend. PCL is considered a superior candidate for this objective, and lignin/PCL blends offer remarkable miscibility, thereby favourable mechanical and biological property, and also enhanced functionality (e.g., by exposure of hydroxyl groups) (Li et al., 2001; Salami et al., 2017). Wang et al. (Wang D. et al., 2019) developed a lignin/PCL blend nanofiber system that could benefit from the functionality of lignin to induce biomineralization and to form an integrated bioactive, osteoconductive bone-mimicking hydroxyapatite (HA) layer on the nanofibers, Figure 3A. Possessing a plethora of hydroxyl groups including aliphatic and phenolic hydroxyl groups, lignin can expose many reducing sites for metal ions, whereby enabling the biomineralization (nucleation and growth) of HA via co-deposition of Ca^2+^ and phosphate ions. The HA-lignin/PCL nanofibrous scaffold offers a bioactive platform that encourages the adhesion and proliferation of osteoblastic cells, Figures 3B–D.

Alginate is a well-known anionic polysaccharide that is derived from seaweed and contains guluronic acid and mannuronic acid units (Homaeigohar et al., 2019). By forming an ionic bond between the carboxylate group of alginate’s backbone and a positively charged crosslinking agent, e.g., Ba^2+^, Al^3+^, Ca^2+^, and Zn^2+^ a hydrogel is created (Shi et al., 2016). Offering desirable biocompatibility, enzymatic degradability, insignificant inflammatory reaction, and chemical flexibility and imitating the 3D structure of extracellular matrix (Kim and Kim, 2014), alginate hydrogels have been frequently applied for the development of tissue engineering scaffolds (Marrella et al., 2017; Reakasame and Boccaccini, 2017) and drug delivery systems (Guarino et al., 2018). Alginate can be coupled with PCL to form blend and core-shell nanofibers with drug delivery ability (Kim et al., 2018). Regarding the latter case, Kim et al. (Kim et al., 2018) devised a core-shell nanofibrous dressing made of PCL (shell) and alginate (core) that could steadily release *Spirulina* as a bioactive material, already incorporated in the core segment. The as-developed dressing material also showed high water uptake capacity.

As a polysaccharide biopolymer, chitosan (CS) is derived from incomplete deacetylation of chitin. It contains many functional amine and hydroxyl groups and thus shows an exclusive polycationic nature and chelating and film-forming properties. As a proven fact, N-acetyl d-glucosamine as a constituent of chitosan drives cell proliferation and hemostasis, thereby accelerating the wound healing process (Keong and Halim, 2009; Patrulea et al., 2015). Chitosan is optimally biocompatible and does not induce adverse reactions when exposed to human cells. Additionally, it is readily biodegraded by enzymes to non-harmful by-products (Chou et al., 2015). Causing prompt blood clotting, chitosan-based bandages and hemostatic products have been approved by the US FDA (Wedmore et al., 2006). Thanks to a non-desirable exceedingly high viscosity of chitosan solutions, their processing via electrospinning is challenging. Moreover, chitosan nanofibers typically show inferior mechanical properties and are unstable when exposed to aqueous media (Cr et al., 2018). As a result, they are commonly blended with biocompatible synthetic polymers such as PCL. For instance, curcumin (CUR)/chitosan (CS) has been blended with PCL as a nanofibrous wound dressing with antioxidant, antibacterial, and cell proliferative effect (Fahimirad et al., 2021). Such blend nanofibers were subsequently surface decorated with electrosprayed curcumin loaded chitosan nanoparticles. According to a variety of biological tests including antibacterial test, *in vitro* cell culture test, *in vivo* wound healing assay, and histological analysis, the hierarchical, blend nanofibers were successful in induction of a wound healing effect in the methicillin-resistant *Staphylococcus aureus* (MRSA) infected wounds. According to the *in vivo* tests, in the presence of PCL/CS/CUR nanofibers, wounds are re-epithelized faster, thereby accelerating the wound closure. In fact, such a consequence enables the wound to be further healed and the damaged skin tissue to be regenerated (Borena et al., 2015). Particularly, PCL/CS/CUR nanofibers that were electrosprayed with CUR/CS nanoparticles provoked the wound healing process, reflected in 96.25 and 98.5% wound healing percentage for the MRSA treated and untreated wounds, respectively.

### Biomolecules (Proteins)

The poor bioactivity and insufficient number of cellular recognition sites (biochemical cues) are major shortcomings of PCL nanofiber mats that lead to lower cellular activities such as cell adhesion, proliferation, and migration, and thus hinder their extensive use in biomedicine (Homaeigohar et al., 2021a). On the other hand, the *in vivo* interaction of the PCL nanofibers is crucial to avoid foreign body reactions such as infection, inflammation, embolization, and thrombosis. To tackle the mentioned bottlenecks and to achieve the desired biological features, PCL has been blended with hydrophilic biomolecules (proteins), e.g., collagen (Lee et al., 2008), gelatine (Anjum et al., 2017; Jiang et al., 2017), bovine serum albumin (BSA) (Homaeigohar et al., 2021a), and silk fibroin (Nazeer et al., 2019) among others.

As a general strategy to improve bioactivity of synthetic biomaterials including PCL, natural polymers with cell recognition sequences are hybridized to modulate the cell-material interactions (Shakesheff et al., 1998; Langer, 2000; Entcheva et al., 2004). Many ECM proteins possess the RGD (arginine-glycine-aspartate) motif, where cells adhere to. The cell adhesion is governed by the interaction of integrins, i.e., cell surface (membrane) receptors to the ligands available in the ECM proteins (Jabbari, 2011). RGD coupled polymers typically show increased cell adhesion and proliferation, as previously validated for fibroblasts (Collier and Segura, 2011) and osteoblasts (Benoit and Anseth, 2005).

**FIGURE 4:**
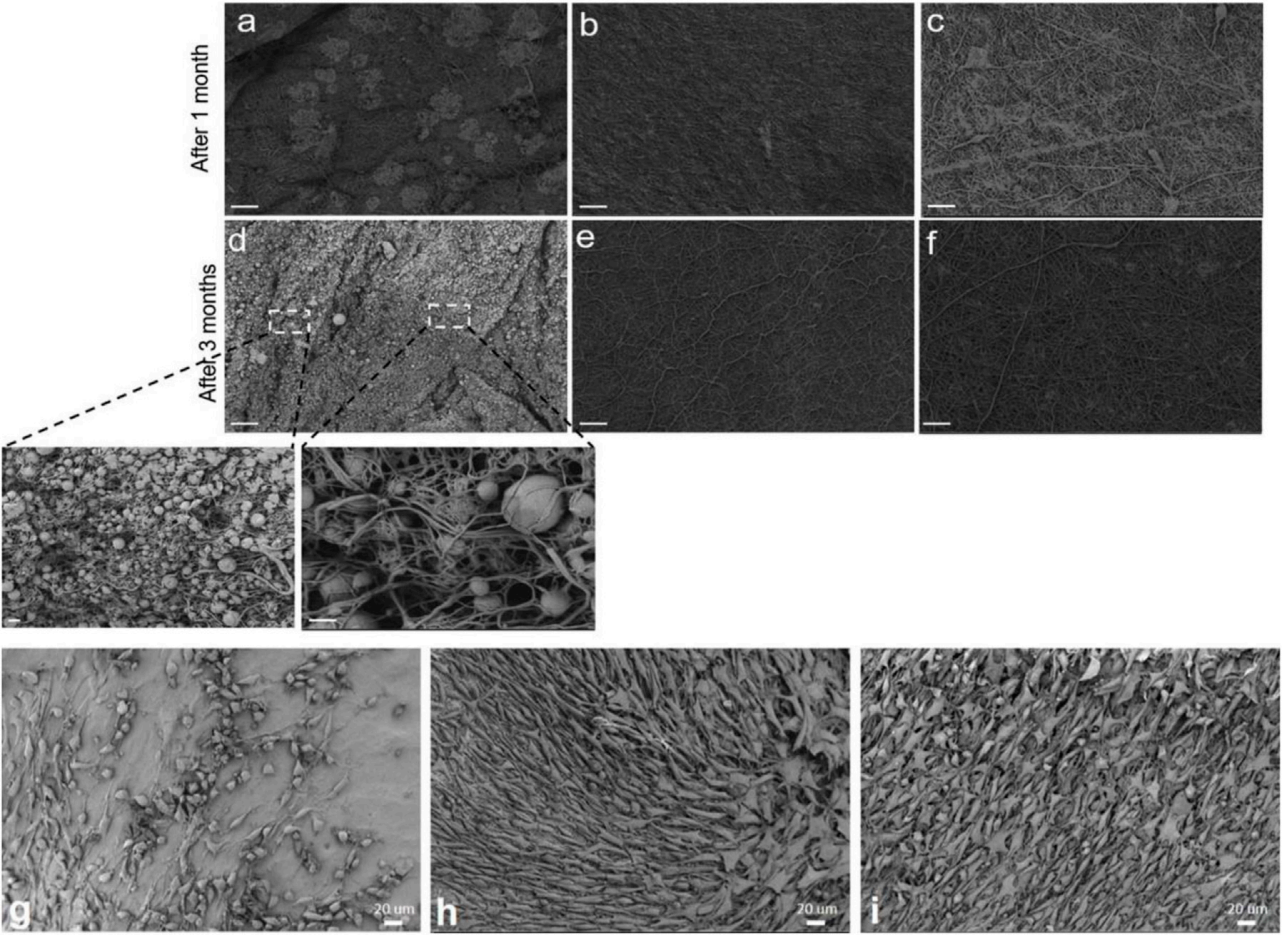
Time dependent hydrolytic degradation process in PBS visualized by SEM images for: PCL nanofibers **(A,D)**, 1 wt% BSA/PCL nanofibers **(B,E)**, and 3 wt% BSA/PCL nanofibers **(C,F)** (scale bars **(A–F)** 20 μm and inset images of **(D)** 1 μm (right) and 3 μm (left)). SEM images represent the NIH 3T3 cell population cultured on: **(G)** PCL nanofibers, **(H)** 1 wt% BSA/PCL nanofibers, and **(I)** 3 wt% BSA/PCL nanofibers, after 4 days. Reproduced with permission. (Homaeigohar et al., 2021a), Copyright 2021, Elsevier.

In a recent study (Homaeigohar et al., 2021a), we enhanced the bioactivity and cell-material interaction of PCL nanofibers by inclusion of BSA. The BSA protein is obtained from cow blood which is an abundant by-product in the cattle industry (Homaeigohar et al., 2020). Therefore, BSA can be used as a commercial, low cost biomolecule for the purpose of biofunctionalization of the PCL nanofibers at a large scale. The BSA/PCL nanofibers were proved to be bioactive as reflected in the formation of inorganic minerals (calcium carbonate) on the nanofiber surface when submerged in simulated body fluid (SBF). Additionally, the formation of hydrogen bonding between the functional groups of PCL and BSA led to a more profound mechanical stability of the BSA/PCL nanofiber mats, as validated through a tensile test as well as a hydrolysis test. As seen in Figures 4A–F, in contrast to the BSA/PCL nanofibers, neat PCL nanofibers were notably degraded after one and 3 months in phosphate buffered saline (PBS). Particularly, after 3 months, PCL nanofibers were crumpled and transformed to spheres and their dominant fraction disappears. The positive impact of BSA is not limited to improvement of structural properties of PCL nanofibers, but biological properties were also enhanced. According to the WST-8 cell viability results, fibroblast (NIH3T3) cells could adequately interact with the BSA/PCL nanofibers and properly proliferated when exposed to them, Figures 4G–I (Homaeigohar et al., 2021a). This finding is promising with respect to the wound healing process wherein fibroblasts play a major role.

Other than blending, surface treatment of PCL nanofibers and their decoration with biomolecules have been also investigated. For instance, the PCL nanofibers surface can be carboxylated through plasma treatment, allowing for adhesion of protein molecules based on a covalent bond between carboxyl and protein (platelet-rich plasma (PRP))’s amine groups (Miroshnichenko et al., 2019). This simple and technologically feasible strategy enables human fibroblasts to adhere, to spread, and to grow on the PRP/PCL nanofibers. Such a biofunctionalized PCL nanofiber system can be potentially employed for wound healing, given the proper, stable immobilization of PRP assuring its long-lasting biological activity, particularly within the harsh medium of wounds (Miroshnichenko et al., 2019).

As a natural protein, silk fibroin (SF)’s biocompatibility has justified its extensive applicability in biomedicine, e.g., pertaining to bone and skin tissue engineering (Koh et al., 2015; Ribeiro et al., 2017; Sang et al., 2018). *Bombyx mori* (*B. mori*) silk fibroin comprises two distinct chain polypeptides in terms of molecular weight (i.e., light and heavy chain with Mw∼26 and 390 kDa, respectively) that are coupled via a disulfide bond (Zhou et al., 2000). The SF fibers are coated with sericin, i.e., a hydrophilic protein (20–310 kDa), that is removed via the degumming process (Inoue et al., 2000). As proven in literature (Nazeer et al., 2019), incorporation of SF in PCL nanofibers can optimize biomechanical properties as well as fibroblast cell proliferation. The latter feature originates from improved hydrophilicity of the PCL nanofibers after inclusion of SF that performs as a cell anchorage site and emanates polar amine and hydroxyl groups. As a proven fact, SF’s RGD sequence positively affects the adhesion of mammalian cells (such as stem cells, fibroblasts, and osteoblasts) (Sofia et al., 2001; Chen et al., 2003).

Gelatine (Gel), as a conventional natural polymer, is synthesized via incomplete hydrolysis of collagen. In comparison with collagen, Gel possesses a lower molecular weight rendering its electrospinning easier. On the other hand, its integrin anchorage zones are abundant, allowing for better cell adhesion, cell migration, and cell differentiation (Nichol et al., 2010). By coupling Gel and PCL, their respective deficiencies are properly addressed (Gloria et al., 2010; Pok et al., 2013), and the Gel/PCL hybrid can be applicable with respect to reconstruction of skin (Chong et al., 2007), tooth (Yang et al., 2010), nerve (Ghasemi-Mobarakeh et al., 2008), and muscle tissues (Kim et al., 2010). The Gel/PCL nanofibers are properly bioresorbable. As mentioned earlier, PCL can be degraded through several mechanisms involving chemistry and enzymatic activities. On the other hand, Gel is degraded by stromelysin and gelatinases (Atkinson et al., 1992), that are released by epithelial cells, fibroblasts, etc., for instance over the course of the inflammatory phase of wound healing and affect cell migration in the proliferative phase (Clark et al., 2007).

**FIGURE 5:**
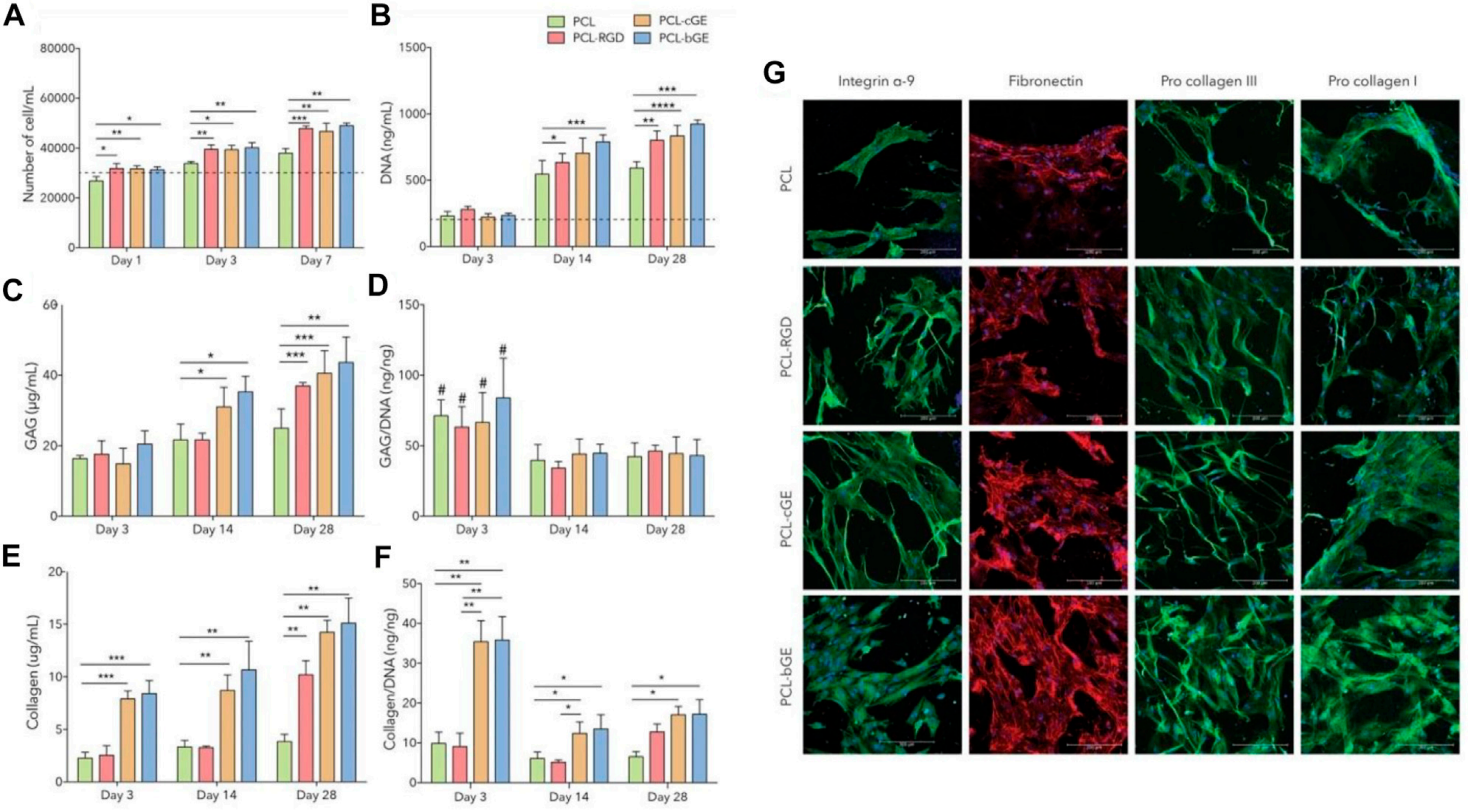
Gel/PCL nanofibers provoke the generation of ECM components by hSKPs. **(A)** hSKPs cell proliferation, **(B)** DNA content of the cells rises over the course of co-culture of the cells and the blended nanofibers implying the impact of Gel and RGD on the cell proliferation. PCL-cGE and PCL-bGE represent Gel coated and Gel blended PCL nanofibers, respectively. In (a&b), the dotted line is representative of primary cell number and DNA content, respectively, **(C)** The amount of GAG secreted in adjacent to different classes of the nanofibers, **(D)** GAG/DNA ratio of the nanofibers (#, i.e., *p* < 0.05 implies a statistically significant difference between day 3 and day 14/day 28, **(E)** The total amount of secreted collagen, **(F)** Collagen/DNA ratio (****, ***, **, and * represent *p* = 0.0001, *p* < 0.001, *p* < 0.01 and *p* < 0.05, respectively). **(G)** Immunofluorescent images show the cellular activities of hSKPs on the Gel/PCL nanofibers, represented by expression of integrin α-9 (green), fibronectin (red), and procollagen I & III (green). Reproduced with permission. (Anjum et al., 2017), Copyright 2017, Nature.

As a skin regenerating material, Gel/PCL nanofibers could stimulate endogenous wound healing and allow for controlled delivery of dermal progenitors (adult human skin-derived precursor cells (hSKPs)). As Anjum et al. (2017) reported, when seeded with adult hSKPs, the composite nanofibers provoke the deposition of sulphated glycosaminoglycans (GAG) and extracellular matrix proteins (Col) (Figures 5C,E). Two distinct classes of Gel/PCL hybrid nanofibers were developed as Gel coated and Gel blended PCL nanofibers for the treatment of skin wounds. In their study, as a control group, PCL nanofiber surfaces were also coated with GRGDS, i.e., a cell binding protein originated from fibronectin, via aminolysis. As compared to the hSKP cells cultured with the neat PCL nanofibers, the total DNA content of those cultured with the PCL-RGD and Gel/PCL nanofibers was at a higher level, implying larger proliferation rate of the cells exposed to the modified and blend nanofibers (Figures 5A,B,D,F). Moreover, the hSKPs seeded on the nanofiber mats secreted fibronectin, i.e., a vital constituent of the ECM that plays a role in the growth of nerve fiber (Figure 5G) (Sakai et al., 2001). The hSKPs also secrete fiber forming Col I and III, that are structural ECM proteins involved in wound healing (Figure 5G). The *in vivo* study also confirmed that after 21 days, all classes of Gel/PCL nanofibers but the blended type could stimulate re-epithelialization and develop a fully epithelialized tissue. Additionally, by immunohistochemical staining of βIII tubulin, it was proved that PCL-RGD and the Gel/PCL blend nanofibers induce formation of nerve fibers that re-innervate a notably larger length of the wound area compared to the gauze control.

Gel/PCL blend nanofibers have also been studied for blood vessel tissue engineering, where inclusion of Gel leads to a more robust system with improved hydrophilicity, in contrast to the neat PCL nanofibers (Jiang et al., 2017). Despite optimum biocompatibility, and enhanced mechanical properties and hydrophilicity, the processing of PCL and Gel combination via electrospinning is challenging due to their phase separation that adversely impacts the nanofiber quality. One solution for this issue could be the use of acetic acid that potentially governs the miscibility of the components, i.e., PCL and Gel, thereby creating homogeneous nanofibers (Feng et al., 2012). Other than blending, VEGF loaded Gel nanoparticles have been also embedded in PCL nanofibers to raise the differentiation rate of mesenchymal stem cell (MSC) and to provoke angiogenesis of endothelial cells (Jiang et al., 2018). The encapsulation of VEGF in Gel particles shields the growth factor molecule against degradation in the harsh physical and biological media within the human body and assures their steady and tailored delivery. Additionally, the Gel nanoparticles endow the PCL nanofibers with larger biofunctional anchor points and thus encourage cell adhesion and proliferation, as reflected in the higher NANOG expression of the cells. In contrast, inclusion of VEGF in Gel nanoparticles downregulated such a factor (along with OCT3/4) in MSCs and upregulated CD31 and vWF quantities, implying its significant contribution to differentiation of MSCs to endothelial cells (EC). Such a system could be proposed as a growth factor delivery platform with potential applications in vascular tissue engineering.

Table 5 tabulates the main blending bioagents that have been investigated as additives to PCL nanofibers for biomedicine.

**TABLE 5:** Bioblended PCL nanofibers for biomedical applications (studies carried out since 2017 have been taken into account).

Blending agent	Nanofiber material	Loading method	Biological effect	Target application	References
Silk fibroin	PCL	Blend electrospinning	Bioactivity	Meniscus regeneration	Li et al. (2020)
Recombinant spider silk protein	Gel/PCL	Blend electrospinning	Bioactivity	Vascular tissue engineering	Xiang et al. (2018)
*m*-RNA	PELCL/PCL-REDV	Emulsion electrospinning	Post-transcriptional gene regulators provoking tissue regeneration	Vascular tissue regeneration	Zhou et al. (2018)
Cartilage derived ECM	PCL	Blend electrospinning	Cartilage regeneration	Cartilage tissue engineering	Feng et al. (2020)
Kartogenin	PGS (core)/PCL (shell)	Coaxial electrospinning	Chondrogenic differentiation promoter and chondroprotective	Cartilage tissue engineering	Silva et al. (2020)
Gel	PCL	Blend electrospinning	Osteogenesis	Guided bone regeneration	Ren et al. (2017)
Synthetic polypeptide	PCL	Blend electrospinning	Antibacterial effect	vascular grafts or wound healing	Liu et al. (2018)
Starch	PCL	Co-axial electrospinning	Enhanced cell viability	Wound dressing	Komur et al. (2017)
Alginate	PCL	Blending and co-electrospinning	Providing a low cell-adhesive platform	*Cancer* stem cells enrichment	Hu W.-W. et al. (2019)
Nanofibrillated chitosan	PCL	Physical blending and electrospinning	Mechanical reinforcement and biocompatibility enhancement	---	Fadaie et al. (2018)
Collagen	PCL	Blend electrospinning	Bioactivity	Corneal endothelium tissue engineering	Hu Y. et al. (2019)
Chitosan	PCL	Blending and co-electrospinning	Improved biocompatibility and antibacterial activity	Skin tissue engineering	Guha Ray et al. (2018)

PELCL, poly (ethylene glycol)-b-poly (l-lactide-co-ε-caprolactone); PGS, poly (glycerol sebacate); REDV, peptide: Arg-Glu-Asp-Val.

### Inorganic Nanomaterials

For particular tissue engineering applications, inorganic bioactive nanomaterials have been added to the PCL nanofibers. For instance, regarding bone tissue engineering, PCL nanofibers alone or as blended with natural polymers, e.g., chitosan, Col, etc. have been reinforced by addition of bioceramics such as hydroxyapatite (HA), tri-calcium phosphates (TCP), bioactive glass, silica (Burton et al., 2019), among others to acquire improved mechanical properties, bioactivity, and even degradability.

The majority of bone tissue engineering scaffolds are based on composite or hybrid materials, developed either by inclusion of hydroxyapatite nanoparticles (nHA [Ca_10_ (PO_4_)_6_(OH)_2_]) and bioactive glass nanoparticles into polymeric materials or via surface deposition (mineralization) of nHA on a polymeric substrate (Sadi et al., 2006; Araujo et al., 2008; Yari Sadi et al., 2008). Considering that nHA is the dominant inorganic phase in bone (hard) tissues, its application as an additive to PCL nanofibers specifically for bone tissue engineering has been frequently explored. nHA shows an exclusive affinity for a variety of adhesive proteins and directly contributes to differentiation of bone cells and also to the mineralization processes (Chen and Chang, 2011). Bone tissue comprises Col type I nanofilaments and hydroxyapatite nanocrystals. Imitating such a structure and composition, nanocomposite scaffolds encompassing bioactive inorganic nanoparticles have shown enhanced bone cell interactions. As validated through *in vitro* and *in vivo* tests, such organic/inorganic composite systems encourage adhesion, proliferation, and differentiation of osteoblasts or MSCs, thereby driving the bone regeneration process (Wang et al., 2007).

**FIGURE 6:**
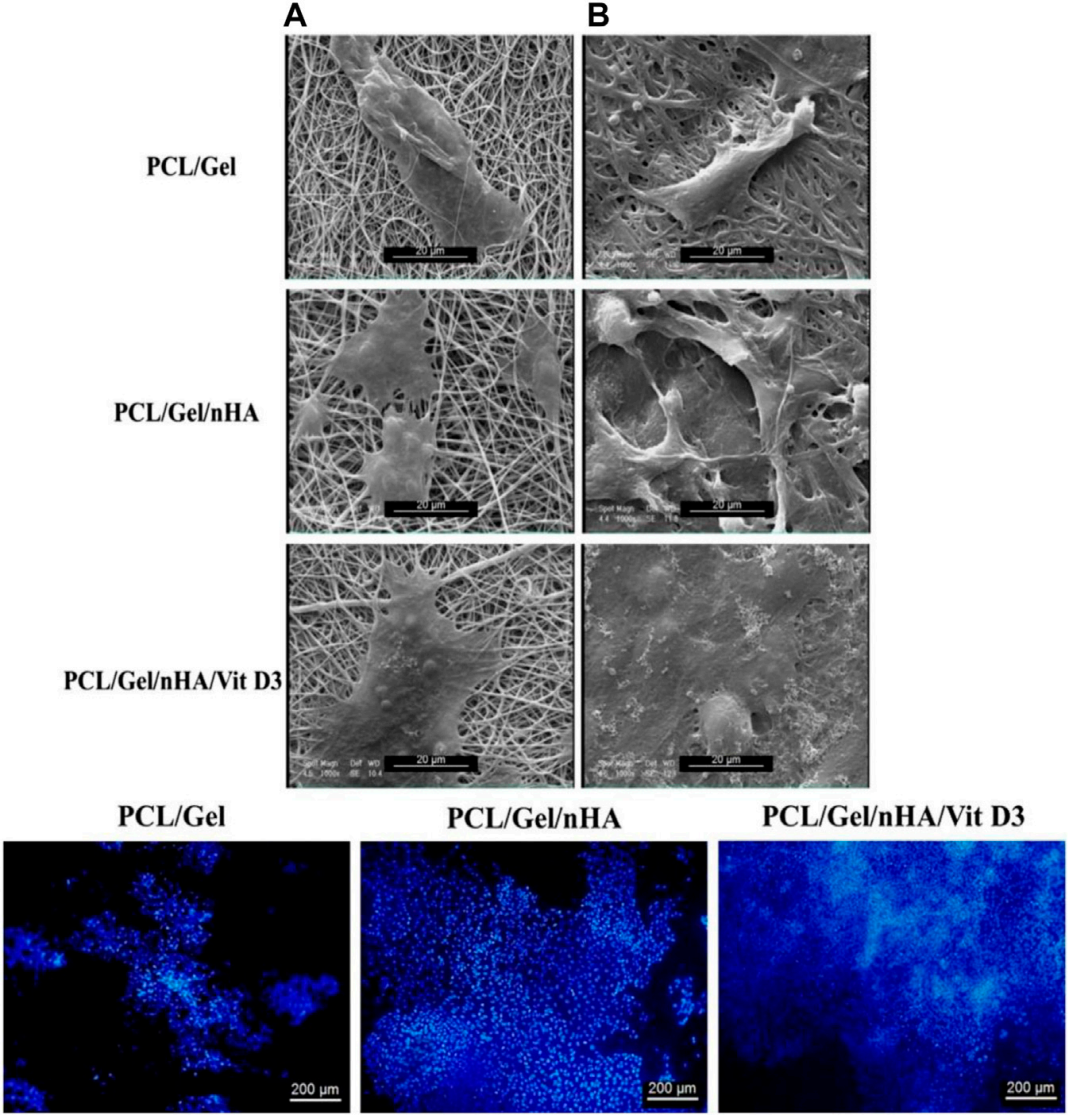
Upper row: SEM images imply the adhesion of MG-63 cells onto PCL/Gel nanofibers with or without hydroxyapatite nanoparticles (nHA) and Vitamin D3 (Vit D3) after 1 **(A)** and 7 **(B)** days cell culture. Lower row: Fluorescence images of the DAPI stained MG-63 cells on the PCL/Gel nanofibers with or without hydroxyapatite nanoparticles (nHA) and Vitamin D3 (Vit D3) after 7 days cell culture. Reproduced with permission. (Sattary et al., 2018), Copyright 2017, Wiley.

To fully mimic bone tissue both in terms of biofunctional composition and structure, it is crucial to develop nanostructured scaffolds with micro/nano-sized hierarchical architecture. By now, biomimicking the native nano-structured bone tissue, comprising Col fibers alongside nHA, has been a sophisticated target in tissue engineering. In this regard, electrospun nanofibers have been appealing due to their extraordinary characteristics such as extensive surface area and mimicry of ECM of native tissues (Yoshimoto et al., 2003; Gautam et al., 2021). PCL nanofibers have been shown to be a suitable platform for bone regeneration and can properly stimulate the differentiation of MSCs and their differentiation to osteoblastic cells (Li W. J. et al., 2005). As an example, very recently, Gautam et al. (Gautam et al., 2021) developed a HA surface deposited Gel/PCL blend nanofiber scaffold for bone tissue engineering. According to the cell viability (MTT) assay and the measured DNA quantities, human osteoblasts were viable and proliferated well on the nanocomposite scaffold. Additionally, the osteoblast cells attached efficiently onto the scaffolds and spread with their specific polygonal morphology. In a similar study, Gel/PCL nanofibers have been reinforced biologically and mechanically by inclusion of HA nanoparticles and Vitamin D3 (Sattary et al., 2018). As shown in Figure 6, SEM images indicate a proper adhesion mode (across a wider area) for the cells (MG63) on the Gel/PCL nanofibers containing HA and Vitamin D3. Additionally, as represented by Fluorescence microscopy images and after DAPI staining of the cells, a higher proliferation rate is observed for the blend nanofibers containing the mentioned additives. The presence of the HA nanoparticles raises the surface roughness of the nanofibers that would promote the cell adhesion.

ZnO is a FDA approved inorganic material with therapeutic effects. ZnO nanoparticles have been embedded in PCL nanofibers to realize a bone/cartilage regenerating composite system with Zn delivery in a controlled manner (He et al., 2021). PCL nanofibers containing ZnO alongside HA are notably effective in terms of angiogenesis and osteogenesis, and can inactivate bacteria and thus prevent infection. As verified via SR-μXRF, Zn is mainly concentrated in the fibrocartilage area of the bone-tendon interface (BTI), implying its likely involvement in the regeneration process of the BTI. Witnessed by Alizarin red staining and picrosirius red staining images, the extent of the putative osteogenic potential of the HA-ZnO/PCL nanofibers was clearly reflected in larger Col secretion and calcium deposition (He et al., 2021). The increased OPN expression, that is a recognized indicator of late osteogenesis, was also monitored to help the identification of early osteogenesis. The therapeutic role of Zn and its contribution to the repair of bone fractures and defects through modulation (upregulating) of TGFβ-SMAD signalling have been already reported (Yu et al., 2017; Rahmani et al., 2019; Shitole et al., 2019). As verified through the Alcian blue staining images, a higher amount of glycosaminoglycans (GAGs) is generated on the nanocomposite nanofibers (He et al., 2021). This finding was in harmony with the immunofluorescence imaging results that clearly verified the improved levels of Col II on the HA-ZnO/PCL nanofibers. According to these results, Zn^2+^ release was reported to promote the release of the insulin-like growth factors (IGF-1), thereby providing better conditions for chondrogenesis (He et al., 2021). IGF‐1 can raise chondrogenesis via enhancement of the proliferation of chondrocytes and the expression of their markers, regardless of TGF‐β signalling (Longobardi et al., 2006). Additionally, Zn^2+^ declines the degeneration of Col via inactivation of matrix metalloproteinases (MMPs) (Toledano et al., 2012).

Silica (SiO_2_) is another inorganic additive to PCL nanofibers that are meant to act as bone tissue engineering scaffolds, for example for nonunion injuries of bone (Burton et al., 2019). Silicon is an element that plays a crucial role in bone growth and mineralization (calcification) during the periosteal ossification processes (Jugdaohsingh, 2007). Lack of silicon has been shown to lead to skeletal deformities in chickens and rats (Carlisle, 1972; Schwarz and Milne, 1972). Burton et al. (Burton et al., 2019) developed nSiO_2_-incorporated PCL nanofiber scaffolds and reported that adult human periosteal cells seeded on such materials find better conditions for proliferation and putative differentiation, eventually leading to the healing of nonunion injuries of bone. As a crucial source of osteoprogenitor and chondroprogenitor cells, periosteum plays a pivotal role in development, growth, and preservation of normal bone or augmentation and repair of injured bone (Colnot et al., 2012). The existence of a healthy periosteum covering the damaged bone segments, particularly in the case of complicated bone damages leading to bone nonunion, is a prerequisite for healing (Ueno et al., 1999). However, rarely the amount of periosteum suffices for bone healing and thus nonunion injuries impose notable clinical burdens (Panagiotis, 2005). To address this bottleneck, engineered nanofiber mats such as nSiO_2_-incorporated PCL nanofiber scaffolds can potentially help the periosteal cells expand and the nonunion injury heal.

The other prominent inorganic additive to PCL nanofibers is bioactive glass (BG) that is meant to raise bioactivity of the composite made thereof. The era of inorganic bioactive materials started with the invention of 45S5 bioactive glass (45S5 Bioglass^®^) by Lary Hench in 1971 (El-Rashidy et al., 2017). This bioactive silicate glass is composed of 45 wt% silica (SiO_2_), 24.5 wt% sodium oxide (Na_2_O), 24.5 wt% calcium oxide (CaO), and 6 wt% phosphorous pentoxide (P_2_O_5_) and as the term “bioactive” implies, BG can form a robust interface with natural tissues as a consequence of a particular biological reaction taking place at the material surface (Hench, 1991). Thanks to the synthesis of a surface layer composed of profoundly reactive carbonated hydroxyapatite, a stable interface with bone (Hench, 1991; Jones, 2013) and even with soft tissues emerges (Miguez-Pacheco et al., 2015). As a crucial feature, BGs can be tailored in terms of chemical composition (Si, Ca, P and Na ions) and ion release capability, thereby stimulating the expression of bone cell genes and thus bone regeneration (Xynos et al., 2001). Inclusion of BG in PCL nanofibers has been diligently pursued by researchers to address the poor cell-matter interaction of PCL. For instance, Luginina et al. (Luginina et al., 2020) incorporated silicate and borosilicate BG nanoparticles in poly (glycerol-sabacate)(PGS)/PCL blend nanofibers to develop a wound healing material. Thanks to the release of pro-angiogenetic ions from the BG particles into body fluid (Day et al., 2004; Gorustovich et al., 2010; Balasubramanian et al., 2018), the as-developed composite nanofiber material can be potentially applied as a wound dressing system. Barrier membranes are conventionally employed for the purpose of guided tissue regeneration (GTR) therapy. One important shortcoming of such systems is their poor bioactivity and inability to induce bone regeneration. To address this challenge, inclusion of osteogenic BG particles in polymeric membranes, e.g. those made from PCL nanofibers, has proven to be an efficient strategy (Hidalgo Pitaluga et al., 2018). Doping of 45S5 BG with beneficial ions such as Sr, Mg, and Zn has been also adopted as a practical approach to provoke bone regeneration by a composite ion substituted BG/PCL nanofiber formulation (Sergi et al., 2020). For example, Sr has shown promising capacity to support osteoblast and osteogenic differentiation, fibroblast proliferation, angiogenesis, and thus to drive osteogenesis (Bonnelye et al., 2008; Gorustovich et al., 2010; Yang et al., 2011; Zhang et al., 2015; Yu et al., 2016; Mao et al., 2017; Weng et al., 2017). Mg also notably contributes to bone regeneration (Cacciotti, 2017), and provokes the proliferation and differentiation of stem cells and impacts on ALP secretion thereby it largely modulates bone metabolism (Dasgupta et al., 2010). Zn is an essential ion that plays a role in the proliferation and growth of cells and is involved in enzyme and growth factor expression, as well as DNA replication (Huang et al., 2017; Maret, 2017). Additionally, Zn ions are potentially able to inactivate pathogens and bacteria (Pasquet et al., 2014). Co-existence of such beneficial ions in the BG phase of BG/PCL nanofibers assures improved wound healing conditions thanks to the higher bioactivity of such nanocomposite nanofibers. Upon exposure to the nanofibers, cell viability (cell adhesion and proliferation) and thus wound-healing rate were reported to raise (Sergi et al., 2020).

### Carbon Nanomaterials

The biomimicry of the ECM, not only in terms of composition but also morphology, is critical to assure the suitability of a scaffold for tissue engineering purposes. Despite having a similar morphology with the Col nanofibrils of the ECM, the electrospun nanofibers’ surface is typically smooth, which does not replicate that of natural nanofibrils. To maximize the biomimicry, thereby influencing cell adhesion and proliferation, it is crucial to develop nanofibers with a periodic nanostructured surface topography as seen in ECM collagen nanofibrils (Fiedler et al., 2013; Wu et al., 2019). In this regard, inclusion of carbon nanotubes (CNTs) into a polymer matrix has been a successful strategy to form a unique structure, called collagen-like nanohybrid shish-kebab (NHSK), wherein fibrous CNTs and polymer lamellae simulate shish and kebab, respectively (Li C. Y. et al., 2005; Li et al., 2006). Wu et al. (2019) extended this idea to electrospun nanofibers composed of CNT and PCL whose shish-kebab structure, developed through a self-induced crystallization approach, could promote osteoblast cell-matter interactions beneficial for bone tissue engineering. The CNT concentration can modulate the mechanical properties of the composite nanofibers and more importantly the frequency, i.e., the repetitive number, of the shish-kebab structure that further impacts the cell behaviour (adhesion and proliferation). Other than the mentioned structural role, CNTs *per se* affect osteoblasts and their interaction leads to bone formation through an enhanced calcification, i.e., a necessary step of *de novo* bone formation (Shimizu et al., 2012). It has been shown that the genes related to osteoblast phenotype are more expressed when exposed to CNT-incorporated nanocomposite films compared to the pristine polymeric ones (Lee et al., 2015). Additionally, CNTs trigger the release of fibroblast growth factors, thereby stimulating the formation of new bone tissue (Hirata et al., 2013). An extra merit of CNTs is their electrical conductivity that enables electrical stimulation through electroactive CNT/polymer (e.g., PLA) nanofibers and thus bone regeneration (Shao et al., 2011). Although CNTs are indeed versatile additives in polymeric scaffolds (e.g., PCL nanofibrous scaffolds), their high cost and relatively notable unit cost restrict their extensive applications (Santhosh et al., 2016; Ghadimi et al., 2020). Furthermore, raw CNTs contain potentially toxic metal catalysts, while those chemically treated are reported to be non-toxic (Chen and Wang, 2006). Therefore, the use of CNTs as additive in polymeric scaffolds for broader clinical translation is only relevant when economical synthesis techniques are available and their potential hazardous effects are minimized either chemically or by creation of less risky alternatives, e.g., carbon nanocrystals (Santhosh et al., 2016).

**FIGURE 7:**
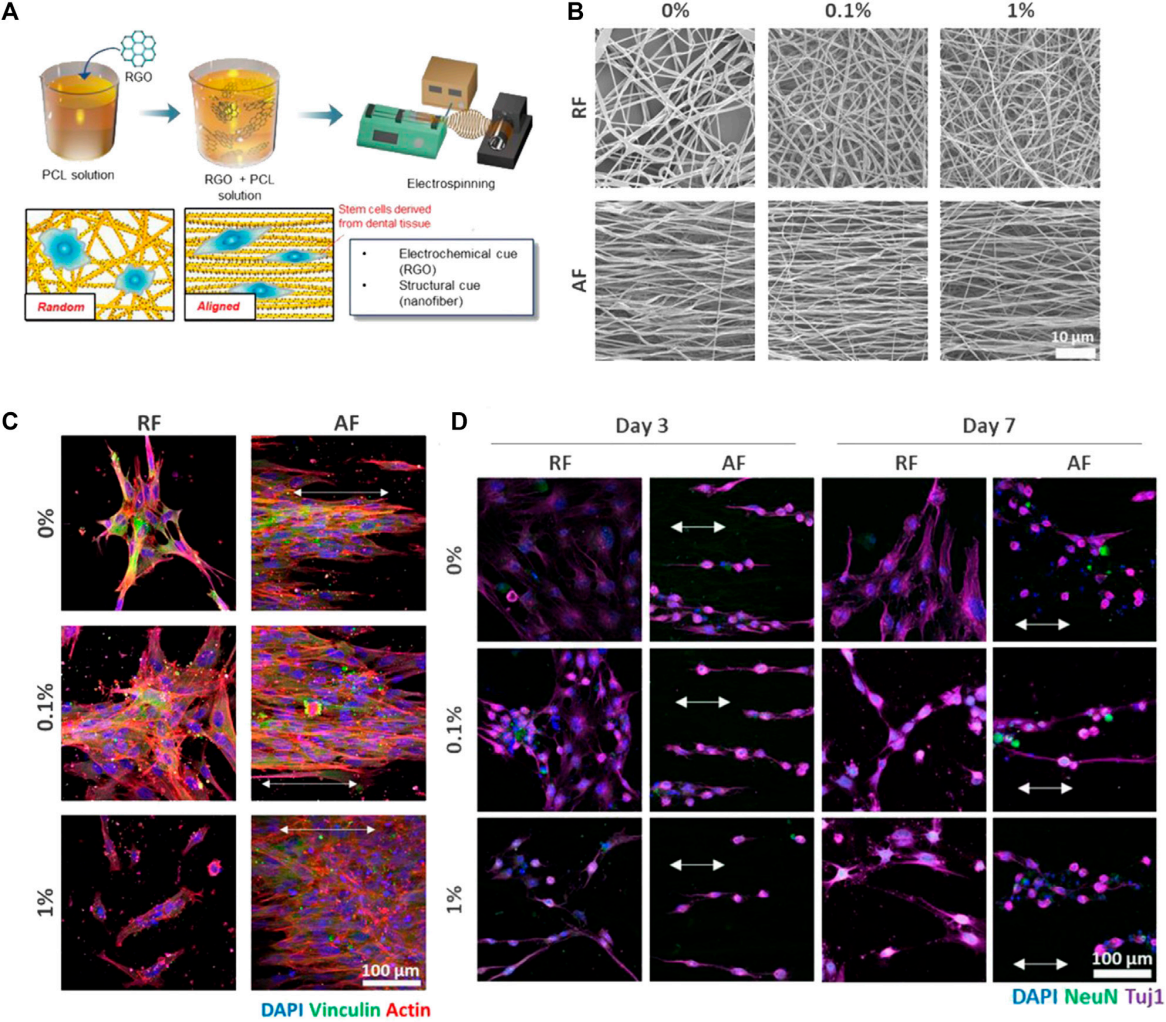
**(A)** The preparation procedure of the rGO/PCL nanofibers and schematic of DPSCs-nanofiber interactions depending on the nanofibers’ alignment mode. **(B)** SEM images show the morphology and alignment of the rGO/PCL nanofibers at different rGO concentrations. **(C)** The immunocytochemistry images taken after 48 h seeding of the cells alongside the rGO/PCL nanofibers imply the interplay between the nanofiber alignment and the cells’ shape. Noteworthy, the cells present on the nanofibers containing the highest rGO filling factor (1%), were not stained with vinculin, which represents focal adhesion. This indicates poor cell adhesion probably due to the adverse effect of rGO at high amounts. **(D)** The immunocytochemistry images imply the neurogenic differentiation of DPSCs induced by the rGO/PCL nanofibers, that encourage morphological transformation in a shorter time and NeuN and Tuji-1 expression at an early stage. The arrows indicate the alignment of DPSCs on the AFs. Reproduced with permission. (Seonwoo et al., 2018), Copyright 2018, MDPI.

As a well-known state of the art additive to polymeric scaffolds/substrates, graphene and its derivatives including graphene oxide (GO) and reduced graphene oxide (rGO) have been appealing for potential applications ranging from biosensors, membranes, and superconductive materials to drug delivery and tissue engineering (Pinto et al., 2013; Lee et al., 2014; Homaeigohar and Elbahri, 2017; Homaeigohar et al., 2021b). In biomedicine, the outstanding potential of graphene stems from its aromatic groups (enabling π-π bonding), polar, reactive functional groups, robust C=C bonding, superior elasticity, and excellent electrical conductivity allowing for its promising interaction with biomolecules, cells (fibroblast, nerve, osteoblast, MSC, etc.), drugs, and tissues (Wan and Chen, 2011; Pinto et al., 2013; Ryu and Kim, 2013; Sayyar et al., 2013; Goenka et al., 2014; Luo et al., 2015). Particularly, thanks to the possibility of π–π stacking and hydrophobic interactions, graphene shows a notably elevated drug holding capacity (Liu et al., 2013). This feature can be employed as the basis of development of graphene based drug delivery systems that can target specific sites and deliver anti-cancer drugs and genes in a controlled manner (Liu et al., 2013). It is worthy to note that graphene nanomaterials can offer a drug loading capacity of up to two folds larger than typical drug delivery systems, e.g., nanoparticles (Davis et al., 2008; Heidari et al., 2019). Another advantage of graphene as an additive to polymeric matrices is its reinforcing role, whereby notably improving the elastic modulus, toughness, and tensile strength of polymer composites (Wan and Chen, 2011; Qi et al., 2013). Additionally, graphene related nanomaterials confer polymer matrices antibacterial activity due to their strong destructive effect on the cell (bacteria) membrane. Considering the mentioned pros of graphene, it has been used as an additive to Gel/PCL blend nanofibers to act as a drug (TCH) holding/delivery component and to raise the mechanical properties and electrical conductivity for neural tissue engineering application (Heidari et al., 2019). The as-developed composite PCL nanofibrous material could kill 99% of Gram-positive and Gram-negative bacteria, thanks to the antibacterial activity of graphene as well as the included drug that was steadily released due to the π–π interaction between graphene sheets and the drug molecules. The underlying mechanism for the antibacterial effect of graphene lies in the fact that free electrons within graphene hamper the multiplication process of prokaryotic cells and as a result inhibit the microbial growth. However, this mechanism poses no risk to eukaryotic cells. On the other hand, upon intimate adhesion of bacteria on the graphene surface, the bacteria’s membrane is subjected to the stress applied by the sharp edges of graphene, leading to physical damage of the membrane and its disintegration (Lu et al., 2012; Heidari et al., 2019). Seonwoo et al. (2018) incorporated rGO in PCL nanofibers as a platform promoting dental pulp stem cells (DPSC) neurogenic differentiation and thus their neurogenesis. In such a system, the PCL nanofibers *per se* provide the cells with structural cues, while rGO enables electrochemical signalling. As a result, cooperatively the nanocomposite nanofibers provoke DPSC differentiation for neurogenesis. Figures 7A,B show the preparation procedure of the rGO/PCL nanofibers and their different alignment modes that could impact cell behavior and morphology. While DPSCs seeded on the randomly aligned fibers (RFs) did not take any particular shape, those located on the aligned fibers (AFs) stretched along the fibers orientation, Figure 7C. Furthermore, the alignment mode was also dependent on the rGO filling factor. The highest rGO amount (1%) led to lower alignment of the cells. In terms of cell proliferation, it was shown that a proper filling factor of rGO as well as the presence of aligned fibers (AFs) notably rise the cell number. Figure 7D demonstrates the interplay of rGO filling factor and fibers orientation with the neurogenic differentiation of the DPSC cells. The morphology of the cells notably alters after 3 and 7 days incubation of the cells with the nanofibers containing 0.1% and 1% rGO. These nanofibers provoke expression of Tuj-1 and NeuN, i.e., the early and late marker of neurogenesis, respectively. Particularly, the NeuN expression was found to be more profound on the nanofibers with the highest rGO concentration or aligned nanofibers.

Table 6 tabulates the main inorganic and carbon nanomaterials that have been investigated as additives to PCL nanofibers for biomedicine.

**TABLE 6:** Inorganic and carbon nanofillers of PCL nanofibers for biomedical applications (the studies carried out since 2017 have been taken into account).

Nanofiller	Nanofiber material	Loading method	Biological effect	Target application	References
Ag NP	Collagen coated PLGA/PCL	Polydopamine based reduction of Ag NPs on polymer blend nanofibers	Antibacterial effect	Alveolar/craniofacial bone regeneration	Qian et al. (2019)
Ag NP	PVP/PCL	Physical blending and electrospinning	Antibacterial effect	---	Li et al. (2019)
GO	Gel/PCL	Physical blending and electrospinning	Cell Proliferation enhancement, mechanical reinforcement, and drug carrier	Neural tissue engineering	Heidari et al. (2019)
Graphene nanoplatelets	PLA-PCL copolymer	Physical blending and electrospinning	Mechanical reinforcement	---	Chiesa et al. (2020)
Bioactive glass NP	Chitosan/PCL	Physical blending and electrospinning	Osteogenesis, angiogenesis, and antibacterial activity	---	Liverani et al. (2018)
Bioactive glass NP	PGS/PCL	Physical blending and electrospinning	Pro-angiogenesis, wound healing effect	Soft tissue engineering	Luginina et al. (2020)
Silica NP	PCL	Physical blending and electrospinning	Mechanical reinforcement and osteoconductivity	Guided bone regeneration	Castro et al. (2018)
Hydroxyapatite NP	Gel/PCL	Physical blending and electrospinning	Mechanical reinforcement and bioactivity	Bone tissue engineering	Sattary et al. (2018)
Octacalcium phosphate	PLGA/PCL	Physical blending and electrospinning	Osteoinductive effect	Guided bone regeneration	Wang Z. et al. (2019)
magnesium phosphate nanoflakes	PCL	Physical blending and electrospinning	Osteoconductivity	Bone tissue engineering	Perumal et al. (2020)

PLGA, poly-lactic-co-glycolic acid; PVP, polyvinylpyrrolidone.

## Conclusion and Outlook

PCL electrospun nanofibers are highly attractive for a plethora of biomedical applications spanning from wound dressing to various tissue engineering scaffolds. Such attractive capacity originates from biodegradability, easy processability, and the relatively low cost of this polymer. However, insufficient interaction with cells, hydrophobicity, and poor mechanical properties of PCL nanofibrous systems that might mismatch with the hosting tissue properties limit their further usability in biomedicine. The main solutions for such shortcomings are either the surface treatment of PCL nanofibers or their hybridization. Thanks to a large specific surface area that can be engineered to encompass many functional groups and to load hydrophilic, bioactive and/or therapeutic agents, PCL nanohybrid nanofibers can turn to a bioactive, hydrophilic nanostructured therapeutic/regenerative platform. On the other hand, inclusion of drugs and therapeutic agents in PCL nanofibers can lead to their controlled release while incorporation of nanofillers can guarantee improved physicochemical properties of the nanofibrous biomedical systems made thereof. Despite the significant progress taken place in the design, engineering, and application of PCL hybrid nanofibers, there are still several gaps that should be targeted in the future by researchers to address and thereby to further develop their potential applications.

A simple glimpse in the literature reflects that applications of PCL hybrid nanofibers have been investigated for wound dressing and engineering of bone, nerve, cornea, vessels, cartilage, and meniscus tissues. Additionally, PCL hybrid nanofibers have been used in relation to cancer research and treatment of periodontal disease (periodontitis). Considering the versatility and high potential of PCL nanofibers, many more applications related to hard and soft tissue engineering can be aimed in the future. One potential area could be electroactive scaffolds that are typically used to restore the performance of destroyed cardiac tissue and in general muscle tissue engineering.

With respect to soft tissue engineering, in addition to surface functionality, PCL nanofibrous systems should support cell anchorage signals mechanically thus enabling the cells to spread largely on the surface. The modulation of surface mechanical properties of PCL nanofibers within the range of soft tissues’ has been overlooked and insufficient attention has been paid to mechanotransduction to govern the cellular adhesion on these electrospun nanofibers. Typically, PCL hybrid nanofibers are highly stiff, while soft tissue engineering requires the nanofiber scaffolds much softer than those currently available. Otherwise, biomechanical disharmony will lead to failure of the tissue regeneration strategy based on such nanofiber scaffolds, as the cell adhesion is directly associated with cell signalling and gene expression of the cells depends on the cell interaction with the substrate.

Synthesis approaches for PCL hybrid nanofibers are limited to a few techniques, mainly blend electrospinning. Co-axial and emulsion electrospinning techniques have been rarely employed probably due to their complexity compared to blend electrospinning. On the other hand, surface functionalization and in general post treatment of PCL nanofibers have been insignificantly taken into account. This might originate from poor functionality of the PCL nanofiber surface that mandates the involvement of supplementary techniques such as plasma oxidization. One significant challenge regarding blend electrospinning, particularly in the case of inclusion of nanofillers, is their entrapment within the nanofibers, rather than their surface residence. This issue definitely restricts the interactivity of bioactive nanofillers with the external medium and thus their efficiency. On the other hand, according to the Coffee stain effect, some therapeutic agents that are highly soluble in the electrospinning solvent might be driven towards the surface, while being loosely bound. This will lead to their burst release in a short time window and adverse toxic effects. The incorporation of delicate, sensitive agents into PCL nanofibers via core-shell electrospinning might be also challenging, considering the high shearing forces and high voltages applied where shell and core fluids meet. Taken together, the synthesis strategy should be properly regulated considering all the involved risks and concerns. One further issue is related to the porosity and pore size of PCL hybrid nanofiber mats that should be maintained properly and not sacrificed against inclusion of additives. As some additives are readily ionized, they can potentially reduce the nanofiber diameter during electrospinning. As a result, pore size can decline notably, cell penetration into the mat could be inhibited and permeability to nutrients/waste and air/water exchange could be exacerbated. On the other hand, some additives physicochemically interact with PCL and thus raise the viscosity of the PCL solution that is being electrospun. Consequently, the nanofiber diameter increases and pore size expands, resulting in less topographical cues and mechanical support for the cells. Conclusively, the formulation of the hybrid fibers should be so designed that does not lead to reduction of structural properties.

In terms of formulation, PCL nanofibers hybridized with nature derived compounds that are inherently biocompatible and offer antibacterial and anti-inflammatory effects seem to be better candidates for biomedical applications compared to those hybridized with synthetic additives. Despite such promising potentials of this class of PCL hybrid nanofibers for biomedicine, certified via many relevant studies, there is no commercially viable product made of them in the healthcare market, to the best of the authors’ knowledge. This gap stems from the likely challenges of scalable co-electrospinning of nature-derived therapeutic agents or natural polymers with PCL, that will necessitate involvement of potentially hazardous cross-linkers and other functional impurities to assure their uniform distribution across the nanofiber. On the other hand, PCL is typically electrospun using organic, hazardous solvents whose trace residual amount in the nanofiber could lead to adverse biological and immunological consequences. This fact is generating increasing research efforts on benign solvents for PCL electrospinning.

To provide an antibacterial effect, PCL nanofibers need to incorporate additives such as antimicrobial peptides, Ag ions, drugs (antibiotics), or nature-derived antibacterial compounds. Recalling the fast-paced emergence of antibiotic resistant bacteria, research is directed towards creation of alternative systems for traditional drug delivery PCL nanofibers. One solution could be the development of AMP functionalized PCL nanofibers. This strategy also prevails over metal nanoparticle loaded PCL nanofibers that could excessively release ions beyond the WHO limits. However, *in vitro* and *in vivo* testing of these hybrid nanofibers in long term studies are still necessary to assure their efficiency over the course of the therapeutic process. Similar consideration in terms of lack of long-term *in vivo* studies can be made for the promising technology of ion doped bioactive glass loaded PCL fibers. As a plausible possibility, the *in vivo* trial study might be unsuccessful. Even so, the negative data and the reason behind failure are as important as the positive data and can help the researchers fine tune their approach and material design to raise the potential success of a given therapeutic strategy.

Taking into account the complexity of tissue regeneration and wound healing processes involving various biological steps such as inflammation, angiogenesis, and tissue remodelling, many proposed systems lack efficiency and are not multipurposed. They target only one particular healing goal and are designed to be interactive in that specific respect, e.g., inflammation, or infection. To fulfil realistic medical needs, the nanofiber system should be able to interact with the body from different standpoints and address various therapeutical needs concurrently. This need could be met through development of multifunctional PCL nanofiber systems that can co-release different therapeutic agents, e.g. drugs, bio-derived compounds, etc. over the therapeutic window.

As other nanofiber systems, scalability of PCL nanofibers with a hybrid formulation for purpose of translation into medical technologies is a crucial hurdle. The production process must be scalable and yet cost efficient to justify the utilization of such nanofiber systems in competition with the commercial counterparts currently available in the medical product market.
